# Supplementary material for: Reducing mortality in acute kidney disease through comprehensive laboratory support: Findings from the ICLATA hybrid implementation study in Zambia
Source: PLOS Glob Public Health. 2025 Oct 14;5(10):e0005342. doi: 10.1371/journal.pgph.0005342 (PMC12520337; doi:10.1371/journal.pgph.0005342)
Supplement: Attachment — Submitted filename: Responses to the editor and reviewers.docx [file pgph.0005342.s004.docx]

**Responses to the editor and reviewers**

Journal Requirements:

1. Please include a complete copy of PLOS’ questionnaire on inclusivity in global research in your revised manuscript. Our policy for research in this area aims to improve transparency in the reporting of research performed outside of researchers’ own country or community. The policy applies to researchers who have travelled to a different country to conduct research, research with Indigenous populations or their lands, and research on cultural artefacts. The questionnaire can also be requested at the journal’s discretion for any other submissions, even if these conditions are not met.  Please find more information on the policy and a link to download a blank copy of the questionnaire here: <https://journals.plos.org/globalpublichealth/s/best-practices-in-research-reporting>. Please upload a completed version of your questionnaire as Supporting Information when you resubmit your manuscript.

RESPONSE: we have done this. Thank you

2. Your current Financial Disclosure states, “The author(s) declare that financial support was received for the research, authorship, and/or publication of this article. This work was supported by the International Federation of Clinical Chemistry and Laboratory Medicine (IFCC)'s Task Force on Outcome Studies in Laboratory Medicine (TF-OSLM) (SKM), Fogarty International Center and National Institute of Diabetes and Digestive and Kidney Diseases of the National Institutes of Health grants R01HL144941 (AK) and 2D43TW009744 (SKM), R21TW012635 (AK and SKM) and the American Heart Association Award Number 24IVPHA1297559 <https://doi.org/10.58275/AHA>. 24IVPHA1297559.pc.gr.193866 (AK and SKM).”. However, your funding information on the submission form indicates that you received funding from “American Heart Association, National Institute of Diabetes and Digestive and Kidney Diseases”. Please indicate by return email the full and correct funding information for your study and confirm the order in which funding contributions should appear. Please be sure to indicate whether the funders played any role in the study design, data collection and analysis, decision to publish, or preparation of the manuscript.

RESPONSE: Please find below the full and correct funding information for our study:

“This work was supported by the International Federation of Clinical Chemistry and Laboratory Medicine (IFCC)’s Task Force on Outcome Studies in Laboratory Medicine (TF-OSLM) (SKM); the Fogarty International Center and the National Institute of Diabetes and Digestive and Kidney Diseases of the National Institutes of Health under award numbers R01HL144941 (AK), 2D43TW009744 (SKM), and R21TW012635 (AK and SKM); and the American Heart Association under Award Number 24IVPHA1297559 (AK and SKM). The funders had no role in the study design, data collection and analysis, decision to publish, or preparation of the manuscript.”

The correct order in which the funding contributions should appear is:

1.	International Federation of Clinical Chemistry and Laboratory Medicine (IFCC) – TF-OSLM

2.	National Institutes of Health (R01HL144941, 2D43TW009744, R21TW012635)

3.	American Heart Association (24IVPHA1297559)

3. Please provide separate figure files in .tif or .eps format.

RESPONSE: we have now provided all figures in .tif

**Responses to Editor:**
This study tackles an important problem which is the lack of necessary laboratory tests to diagnose kidney disease and how its lack increases the risk of poor outcomes.

In addition to the comments by the 2 reviewers there some additional concerns and issues that should be addressed:

1. How was the study funded and how much would all the tests cost

RESPONSE: The intervention (comprehensive lab testing) was supported by the International Federation of Clinical Chemistry and Laboratory Medicine (IFCC)'s Task Force on Outcome Studies in Laboratory Medicine (TF-OSLM). The funding was moderate and provided All necessary reagents and test kits as part of the study, at no charge to the patients. We also acknowledge the cost implications of offering a full panel of tests. While we did not perform a formal cost analysis, we estimate that providing the complete lab profile (covering kidney function, liver function, blood counts, infection screens, and additional tests mainly specific etc.) cost on the order of tens of US dollars per patient, roughly $50–$100, varying by tests for the baseline and about $30-50 for follow-up tests. The cost and number of repeated tests varied per patient and were specific for each condition the patients had. We have emphasized that these costs are modest compared to expensive interventions like dialysis or prolonged ICU care that early diagnostics might help avoid. In addition, the funds could not cover other expenses Furthermore, we have added in our Conclusions that future research should examine the cost-effectiveness of this intervention, to directly address this concern.

2.	Were all tests done in each patient? Many of these tests are not always necessary to diagnose AKI; the choice of tests should be based on clinical suspicion. If this was the case, it needs to be explicit. How many and which tests were ordered per patient? How often were tests done – only on admission or throughout the hospital stay?

**Response:** We agree that not every test is needed for every patient, and we have clarified this in the Methods. In the Intervention Cohort (IC), the full laboratory panel was made available to the treating physicians, but tests were ordered according to clinical judgment for each patient’s situation. For example, all IC patients received core investigations (serum creatinine, urea, electrolytes, etc. at baseline) to confirm AKI/AKD and assess severity. Additional tests (like hepatitis serologies, autoimmune markers, cardiac enzymes, etc.) were requested only if clinically indicated by the patient’s presentation. We have made this explicit by stating that the comprehensive test profile was patient-specific: not every single test on the list was done universally, but rather the intervention ensured that whichever tests were deemed necessary for a given patient could be obtained without delay. Regarding frequency of testing, we also clarify that laboratory tests were performed at admission (baseline) and then repeated during the hospital stay as needed. For instance, renal function tests were often monitored daily or every other day in unstable patients, and other investigations were repeated based on clinical evolution (e.g., follow-up hemoglobin after transfusion, repeat cultures if fever persisted, etc.). In the revised manuscript, we describe that the IC had continuous laboratory support throughout hospitalization, not just a one-time test, enabling ongoing assessment. We did not tabulate the exact number of tests per patient (as this varied widely depending on complexity of the case), but we have added qualitative description to assure readers that testing was tailored and iterative rather than one-size-fits-all.

3.	What was the duration of follow-up?

**Response:** We apologize for not making the follow-up period clear in the initial manuscript. We have now specified the follow-up duration in our Methods. The primary outcome was 30-day mortality, meaning we monitored survival status for 30 days after admission for each patient (regardless of whether they had been discharged earlier). Additionally, for secondary outcomes like progression to chronic kidney disease (CKD) or end-stage renal disease (ESRD), as well as rehospitalization, we collected data for up to 3 months after the initial AKD diagnosis, in line with the 90-day definition of acute kidney disease. In the prospective IC, patients were followed through outpatient visits or phone calls up to 90 days (and in a few cases beyond, up to ~6 months for those who consented to extended follow-up) to document longer-term outcomes. We have clarified in the revised text that the IC participants were followed until July 31, 2024, with a median follow-up of approximately 3 months, whereas the retrospective NIC data were limited to what was documented in their records (typically up to discharge and any return visits within 3 months). Thus, the follow-up for mortality was 30 days for all, and for other outcomes up to 90 days, which is now clearly stated.

4.	How were the 39 NIC cases with AKI determined? It is likely these cases had laboratory testing to make these diagnoses… if yes, then what was different between these cases and the IC cases? Please explain, as this is critical to the validity of the entire manuscript.

**Response:** We appreciate this query and have elaborated on it in the Methods. The 39 patients in the Non-Intervention Cohort (NIC) were identified retrospectively from hospital records as having a diagnosis of AKI/AKD. Indeed, those patients must have had at least some basic laboratory testing (particularly serum creatinine) done during their admission in order to recognize AKI/AKD. We clarify that during the NIC period, the hospital’s lab could perform a very limited set of tests – for example, a serum creatinine test might have been done for these patients (when reagents were available) which flagged kidney injury. The critical difference is that in the NIC, beyond such sporadic or minimal testing, there was no comprehensive or timely lab support. Many of the NIC cases had only one creatinine value or a few basic labs over the course of their illness, and often no tests to identify the cause of AKI/AKD were available. In contrast, the IC patients received the full spectrum of relevant tests promptly at presentation and during follow-up, allowing thorough evaluation. We have explicitly stated in the revised manuscript that NIC patients were diagnosed clinically and with whatever few tests were on hand, whereas IC patients had the benefit of complete diagnostic workups. This explanation underlines that NIC’s “lack of necessary lab support” does not mean absolutely zero tests were done; rather it means essential diagnostics were often lacking or delayed. We agree this distinction is crucial for validity, and we have made it clear that the NIC’s AKI diagnoses were made with very limited data (sometimes just a high creatinine and clinical judgment), whereas the IC diagnoses and management benefited from an extensive lab panel.

5.	What does a “prompt manner” mean in terms of the intervention in the IC? How was this different from the NIC?

Response: In the context of our study, “prompt manner” refers to the speed and immediacy with which lab results were obtained and made available to clinicians in the IC. We realized this phrasing was vague, so we have replaced it with a more concrete description. Specifically, we explain that in the IC, laboratory tests were performed and resulted without delay – typically within a few hours on the same day of ordering, in contrast to the NIC era where tests (if available at all) often had significant delays (24–48 hours) or were not done until a crisis arose. In practice, for the IC patients, our study team coordinated closely with the laboratory to ensure expedited sample transport and processing; for example, blood samples from IC patients were prioritized and run immediately on the analyzers, and results were relayed to the treating team as soon as they were ready. In the NIC, by contrast, clinicians frequently had to wait for reagents to be procured or batch testing to be done, leading to diagnostic and treatment delays. We have highlighted these differences in the revised Methods, so it is clear that “prompt” means substantially faster turnaround times and no waiting for critical test results in the IC.

6.	Page 6 line 176 – anemia does not cause AKI, but malaria itself does.

**Response:** You are correct, and we appreciate this clarification. In the original manuscript, we had listed “severe anemia” as a suspected underlying cause of AKD for some patients. We agree that anemia per se is usually a consequence or a co-morbidity, not a direct cause of acute kidney injury. In the revised version, we have corrected this. We identified that those cases of “severe anemia” were in fact associated with malaria (a common cause of both hemolytic anemia and AKI in our setting). Therefore, we have reclassified “severe anemia” as a cause to reflect the likely underlying etiologies, such as malaria. In the Results (Table 1 and text) we now clarify that malaria (with resultant severe hemolysis and anemia) was the actual precipitant of AKI in those cases, rather than implying anemia alone caused AKI. We have also adjusted the discussion to avoid that misstatement. Thank you for pointing this out; the manuscript now accurately attributes AKI to appropriate causes (e.g., severe malaria infection rather than just “anemia”).

7.	Many proportions are stated in the text which are repeated in the table. These do not need to be repeated in the text.

**Response:** We have revised the Results section to eliminate unnecessary repetition of numerical data that is already presented in the tables. In the original draft, we admittedly provided a very detailed narrative of all proportions and p-values, which duplicated the content of the tables. In the updated manuscript, we have streamlined the text: we now focus on key findings and significant differences in the narrative, and we refer the reader to the tables for the full set of numbers. For example, instead of exhaustively listing every percentage, we highlight particularly noteworthy comparisons (such as those with significant p-values or those relevant to our hypotheses) in the text, and avoid restating every figure that the table displays. This makes the text more readable and avoids redundancy, while the tables retain all detailed data for reference.

8.	Line 178: “The proportion of pyelonephritis in the IC compared to the NIC significantly reduced by 100% (p < 0.0001)” is confusing… Rather word this as “was lower” because the testing itself did not “reduce” the incidence of pyelonephritis.

**Response:** We agree that the original wording was misleading. We have rephrased that statement for clarity. In the revised Results, we now state the finding in neutral terms, for example: “Acute pyelonephritis was documented in 11.5% of NIC cases (9 out of 39) but in 0% of IC cases (0 out of 39), p<0.0001.” In other words, we describe it as a difference in observed frequency, rather than saying the incidence was “reduced” by the intervention. This wording makes it clear that pyelonephritis cases were lower in the IC group, without implying a direct causal reduction by testing. We have applied this change not only to the specific line mentioned but wherever similar phrasing issues occurred.

9.	Throughout the manuscript, please avoid the implication that testing “reduced” or “increased” certain diagnoses. The testing increased the chance of diagnosis, and the observed frequency of a diagnosis was higher or lower – but the testing itself did not directly reduce or increase incidence. These statements are confusing and require significant rewriting. Please proofread carefully to make these changes throughout.

**Response:** We appreciate this general guidance and have taken it to heart. We performed a thorough proofreading of the manuscript and rewrote numerous sentences to remove any unintended causal implications. Our revised text now clearly distinguishes between finding a diagnosis more often versus truly changing the patient’s underlying incidence of conditions. For example, instead of saying “the intervention reduced misdiagnosis,” we now say “misdiagnoses were less frequent in the IC than in the NIC.” Likewise, rather than “increased CKD diagnosis” (which we used initially), we clarify that the IC had a higher rate of CKD being identified or recorded, likely due to more thorough evaluation. We ensure the language attributes changes to detection and documentation in the IC, not that testing per se magically changed the disease occurrence. These edits have been made in all relevant sections of Results and Discussion to eliminate confusion.

10.	Similarly, outpatient and inpatient “cases” did not really increase or decrease… The testing may have reduced hospital admissions – how was this determined? Were things “ruled out” by getting faster laboratory results?

**Response:** We have rewritten the statements regarding outpatient vs. inpatient proportions. We now simply report that a greater proportion of IC patients were managed as outpatients, whereas more NIC patients were inpatients at the time of diagnosis, and we avoid saying “increased” or “decreased” as if it were an outcome. We also added an explanation in the Discussion to address the possible reasons behind this observation. We acknowledge that we did not directly measure the decision-making process for admission, but it is plausible that with rapid lab results in the IC, clinicians could quickly rule out certain severe conditions or stabilize patients, allowing some to be treated without admission. For instance, an IC patient with borderline creatinine and no serious complications, once fully evaluated, might have been managed at home with close follow-up, whereas in the NIC era the uncertainty might have led to admission. We mention this hypothesis in the discussion: that prompt diagnostics possibly averted some hospital admissions by providing clarity and enabling timely outpatient interventions. We have been careful to label this as an interpretation, given we did not run a formal analysis on admission decisions. We also corrected the terminology by referring to “patients” rather than “cases” in this context (and throughout the manuscript, as per comment #11).

11.	Please refrain from describing patients as “cases.”

Response: We apologize for the use of impersonal terminology. In the revised manuscript, we have systematically replaced the word “cases” with “patients” (or “participants” where appropriate) when referring to individuals in the study. We agree that this is more appropriate and reader-friendly. The only time we use “cases” now is in a generic epidemiological sense (e.g., “cases of AKI” in the population), but not when referring to our study subjects directly. This change has been implemented throughout the text.

12.	The statement that the testing was beneficial for outcomes may be true, but this would only be the case if it led to interventions and treatments.

**Response:** You are absolutely right, and we have clarified this point. In several places in the Discussion, we now explicitly state that it is the clinical actions taken in response to test results that ultimately improved outcomes. For example, we mention that the availability of comprehensive diagnostics enabled earlier or more accurate identification of the cause of AKI, which in turn allowed timely and appropriate interventions (such as targeted antibiotics for infections, stopping nephrotoxic drugs, urgent dialysis for confirmed acute renal failure, etc.). We have avoided any language that implies lab tests alone (in isolation) improved mortality or recovery. Instead, we emphasize a logical chain: better diagnostic information → better-informed treatment decisions → improved patient outcomes. We have also added a sentence acknowledging that simply providing test results is only beneficial insofar as it changes management – a point which our study supports, since the IC’s improved outcomes presumably reflect more effective treatments guided by those test results.

13.	It seems there was more death in the IC cohort from Table 1 – this is not a better outcome? This appears opposite to what is stated in the text in several places. Please recheck the numbers in the tables and the statements in the text. A Kaplan-Meier curve would be of interest.

**Response:** Thank you for highlighting this, as it uncovered a critical presentation issue. We have double-checked all mortality figures and corrected the text and tables to ensure consistency. We also had to verify the records and have since updated and made a few corrections.To clarify: in our data, the IC had substantially fewer deaths than the NIC, not more. The confusion may have arisen from how the data were presented. In Table 1 of the revised manuscript, we show that 11/39 patients died in the NIC (28.2% mortality) compared to 2/39 in the IC (5.1% mortality). We have made sure the text explicitly reflects this: “Mortality was significantly lower in the intervention group (5.1%) compared to the non-intervention group (28.2%, p=0.012).” We also heeded the suggestion to include a Kaplan-Meier survival analysis. We have now added Figure 3, which is a Kaplan-Meier curve comparing survival between NIC and IC over the follow-up period. This figure vividly illustrates the mortality difference, and we describe it in the Results: the survival curves diverge early, and the hazard of death was more than 8-fold higher in NIC than IC (log-rank p=0.0014). We believe this addresses the concern – the outcomes in IC are indeed better (lower mortality), and both the numeric data and the new figure support the statements in the text.

14.	How was adequacy and timeliness of delivery of results measured? (Line 197)

Response: We acknowledge that we did not formally quantify the turnaround time of lab results in this study. In the original manuscript, we had made a statement about improved “adequacy and timeliness” of results in the IC, but we agree that without concrete metrics this claim could be problematic. In the revision, we have removed any suggestion that we measured timeliness as a numeric outcome. Instead, we qualitatively describe the process improvements (as also detailed in our response to comment #5 above): that in the IC, results were made available swiftly, whereas in the NIC there were delays. We stop short of claiming a measured outcome for this. We focus our results on clinical outcomes and diagnostic yield, rather than process metrics we didn’t formally record. We do mention in the Discussion that one of the presumed benefits of the intervention was rapid turnaround of labs, but we label this as an aspect of the intervention implementation rather than a result we analyzed. In summary, we no longer imply we have a measured “timeliness” outcome; we simply note qualitatively that the IC had faster lab result availability by design.

15.	Table 1 is very long; consider breaking it into two tables, with demographics and clinical findings separated.

**Response:** We agree that the original Table 1 was quite large and could be more reader-friendly if split. With the removal of some variables, table 1 is more concise now. We trust that this change improves the clarity and presentation of our data as suggested.

16.	How was Acute Kidney Disease defined? AKI is not in the table.

**Response:** We realized that our initial manuscript did not clearly define “acute kidney disease (AKD)” up front, which could confuse readers. We have now added a detailed definition in the Methods section. We explain that we used the emerging KDIGO definition of AKD as acute or subacute impairment of kidney function lasting >7 days up to 3 months, which includes but is not limited to AKI. We also define AKI using the standard KDIGO criteria (creatinine or urine output changes) and clarify that AKI is essentially a subset of AKD (usually representing the first 7 days of acute injury). In our study, all patients met criteria for AKD by virtue of having ongoing renal dysfunction in the acute/subacute timeframe, and many of them also fulfilled the AKI criteria at presentation. We did not list “AKI” as a separate variable in the tables because the inclusion was based on AKD (which, again, encompasses AKI). However, to avoid confusion, we explicitly state in the Methods or Results that the majority of patients did present with an acute rise in creatinine consistent with AKI, but some had subacute presentations that still fell under AKD. In short, the manuscript now provides a clear definition of AKD (and how it relates to AKI), and explains that we focused on AKD to capture the full continuum of acute renal impairment, whether or not the strict 7-day AKI criteria were met. We believe this addresses the query and ensures readers understand our use of terminology.

17.	What does “cost of medication” in Table 1 refer to? Why is this relevant?

Response: The “cost of medication” in Table 1 was an exploratory variable we had collected, representing the total in-hospital drug expense per patient (in USD). Our initial thought was that the intervention might influence treatment decisions in a way that affects medication usage (for example, more targeted therapy might reduce unnecessary medications, or conversely, identifying a diagnosis might lead to more medications for that specific condition). However, we agree that this variable was not central to our study questions and caused more confusion than insight. In the revised manuscript, we have removed the ‘cost of medication’ row from the table and omitted it from the analysis/discussion. We decided that without a full pharmacoeconomic analysis, a single line about medication cost (which did not significantly differ between groups) is not particularly informative. By removing it, we keep the focus on clinically meaningful outcomes. We do, however, mention in passing in the Discussion that the intervention did not substantially change medication costs (as an aside to reassure that providing lab tests did not drive up treatment expenses), but this is only noted qualitatively. The primary content about medication cost has been dropped as per your suggestion.

18.	Table 2 is confusing with lumping CKD, ESKD and death all together. Please separate these. Is dialysis available to all who develop ESKD in your setting? If not, this must be stated, as this likely contributed to deaths among those with that diagnosis.

Response: We appreciate this comment and have addressed it on two levels. First, regarding presentation: we realized that in the original submission, our Table 2 and Table 3 might have mixed multiple outcomes together, which was confusing. We have since restructured the way we present outcomes. Chronic kidney disease (CKD) and end-stage renal disease (ESRD) outcomes are now shown separately from mortality in the tables and are also discussed separately in the text. We ensure that death, progression to ESRD, and other outcomes are not all jumbled together but considered distinctly. For example, our revised Table 2 (outcomes table) lists how many patients progressed to ESRD, how many died, etc., in separate rows, making it easy to distinguish these outcomes.

Second, regarding dialysis availability: this is a very important contextual factor, and we have added a note about it in our Discussion (Limitations). In our setting (Livingstone, Zambia), chronic dialysis resources are available but extremely limited. Not every patient who progresses to ESRD can receive long-term dialysis often due to resource and access constraints and there are no kidney transplant services. We now explicitly state that fact. We explain that this limitation likely impacted survival: patients in either cohort who ended up with ESRD had a poor prognosis largely because only a few could continue dialysis beyond the acute period (for instance, some might get acute dialysis during hospitalization, but long-term maintenance dialysis is not guaranteed for all). We agree that the higher mortality among those with a diagnosis of ESRD is partly due to the lack of available definitive renal replacement therapy. By including this explanation, we give readers a crucial context for interpreting the outcomes. it underscores that progression to ESRD in our environment is often fatal not necessarily because of the intervention, but because of systemic resource limitations.

19.	There is mention that much data was missing in the NIC group – please explain exactly where and when data was missing and how many were lost to follow-up. These numbers may have impacted/biased the statistical conclusions.

Response: We have expanded on the issue of missing data and loss to follow-up in the revised manuscript. In the Methods (under a “Bias” or “Data quality” subsection), we detail the following: During the retrospective chart review for NIC, we initially identified 1,136 patient records of interest (e.g., those with kidney-related diagnoses or who had creatinine measured). However, the vast majority of these records did not have complete data needed for our analysis – for example, many lacked documentation of baseline creatinine, or outcome at discharge, etc. In the end, only 39 patients met our inclusion criteria of definite AKI/AKD diagnosis with complete data on key variables and outcomes. We acknowledge that this indicates a lot of missing information in the NIC period. Essentially, many potential AKI cases in NIC could not be analyzed due to missing lab results or follow-up information, and thus were not included. We have now explicitly stated how many records were excluded due to missing data (which is the difference between 1,136 screened and 39 included – the remainder were excluded either because they weren’t AKI or because critical data were missing).

Additionally, we discuss the follow-up: in the NIC retrospective data, post-discharge follow-up was generally not available in the records unless the patient was readmitted to the same hospital. This means some NIC patients may have died or progressed to ESRD after leaving the hospital, but we would not have records of those events (leading to under-ascertainment of those outcomes). We consider the potential bias this introduces: the NIC outcomes might appear better in some respects simply because of incomplete follow-up data (for example, fewer recorded ESRD cases in NIC could be partly due to patients not returning for us to document progression). We mention this in the Limitations, noting that our comparisons must be interpreted with caution given the disparate data completeness. In summary, we now clearly explain where data were missing (chiefly in the NIC charts) and how that could affect the results. We took steps to minimize bias by excluding incomplete records, but we acknowledge the limitation that this may have selected for more severe or well-documented NIC cases, potentially biasing comparisons in unknown ways.

20.	Please explain what is meant by missed diagnosis and delayed diagnosis.

Response: We realize these terms needed definition. In the revised manuscript, we have included an explanation of “misdiagnosis or delayed diagnosis of AKI/AKD.” This was a combined endpoint we assessed, based on chart reviews. Specifically, we define “misdiagnosis” as cases where the patient’s acute kidney injury was initially not recognized or was incorrectly attributed to another condition. For example, a patient might have been first diagnosed with “gastroenteritis” or “malaria” and only later (after labs) correctly diagnosed with AKI – that would count as an initial misdiagnosis of the AKI. Similarly, “delayed diagnosis” refers to cases where the AKI/AKD was eventually identified, but only after a significant delay (e.g., not until several days into admission, when earlier recognition would have been possible if tests had been done). We determined these by reviewing the sequence of notes in the records: if the first physician note did not mention AKI (when in retrospect it was present) or if the AKI was only documented after a lab result came back late, we flagged it as a delayed or missed diagnosis. In simpler terms, the NIC had many patients whose AKI or AKD was only diagnosed late or after transfer to our unit, whereas the IC had far fewer such instances because labs were done immediately. We have added this clarification in the Methods section and also provided examples in the Discussion to illustrate what constituted a misdiagnosis or delay. We agree that defining these terms is important for readers to understand our findings that misdiagnosis/delay was more common in NIC.

21.	How was pre-existing CKD determined? (Stated in the discussion.)

Response: We have clarified in the Methods how we identified and defined pre-existing chronic kidney disease (CKD) in our cohort. Since this was an AKI/AKD study, many patients had a combination of acute and chronic kidney impairment. We defined CKD based on KDIGO guidelines (eGFR <60 mL/min/1.73m² for >3 months, persistent albuminuria, or other markers of chronic damage). In practice, determining pre-existing CKD for our patients involved several approaches:

•	Medical history: We reviewed each patient’s record for any indication of known kidney disease prior to the acute illness (for example, mention of previously elevated creatinine, longstanding diabetes or hypertension with renal complications, or a diagnosis of CKD in the past).

•	Baseline creatinine: In some cases, we estimated whether CKD was likely by using a baseline creatinine (if available from prior outpatient visits) or by assuming that if the admission creatinine was very high and did not fully normalize, chronic disease might be present.

•	Imaging and chronic changes: Some patients had renal ultrasounds or other exams suggesting chronic changes (small echogenic kidneys), supporting a CKD diagnosis.

•	Follow-up: For the prospective IC, we were able to observe whether kidney function recovered by 90 days. If it did not recover to normal, that implied underlying CKD. For NIC patients, if their discharge summary or follow-up documented CKD, we recorded that.

We added a note in the Discussion and Methods stating that pre-existing CKD was identified either through past history or lack of renal recovery, and that our data likely underestimates CKD in NIC due to limited follow-up. We felt this detailed explanation was important, since we reported a higher proportion of CKD in the IC, which we interpret as better detection (many of those CKD cases were newly uncovered because of comprehensive testing). Now the reader will understand the basis on which we called a patient “CKD” in addition to AKD.

22.	The use of nephrotoxic drugs did not differ between NIC and IC. How is this explained?

Response: We have added a brief explanation for this finding in the Discussion. Both NIC and IC had a substantial fraction of patients (~38%) with exposure to nephrotoxic drugs (such as NSAIDs, certain antibiotics, or traditional herbal remedies known to affect kidneys). The fact that this did not differ between cohorts likely reflects the nature of those exposures: many occurred before hospitalization or outside the immediate control of the treating team. For example, some patients developed AKI/AKD because they took NSAIDs or herbal medications at home before admission – this would be equally likely in the NIC period and the IC period. Additionally, during hospital management, it is standard practice (regardless of the intervention) to avoid known nephrotoxins in patients with kidney injury, so we would not expect the intervention to change the prescribing of such drugs; clinicians in both cohorts were presumably cautious about nephrotoxic medications. We also consider that in our setting, the availability of certain drugs didn’t change between the two periods, so the likelihood of exposure remained similar. In short, the intervention was aimed at diagnostics, not directly at modifying nephrotoxin exposure, so it’s not surprising that this risk factor was similar in both groups. We have articulated this reasoning in the revised discussion to satisfy the reviewer’s query.

23.	I disagree that pyelonephritis is likely hospital acquired; this explanation does not seem plausible.

Response: Thank you for this critique. In the earlier version of our discussion, we speculated that perhaps the pyelonephritis cases seen in NIC (and none in IC) could have been hospital-acquired infections in NIC. After reconsideration, we agree that this was not a well-founded explanation, especially given the data. We have removed the suggestion that pyelonephritis was likely hospital-acquired. Instead, we offer alternative interpretations for why pyelonephritis was observed only in NIC: for instance, it could be due to the small sample size (perhaps by chance those infections didn’t occur in the IC sample), or more intriguingly, it might indicate a difference in diagnostic labeling (in NIC, without thorough investigation, a patient with fever and AKI might have been labeled as “pyelonephritis” based on clinical picture, whereas in IC, better diagnostics might have revealed the true cause – e.g., maybe those IC patients were found to have something else, like glomerulonephritis, instead of assumed pyelonephritis). We discuss that possibility. But we do so without implying any hospital-acquired aspect. We also acknowledge that we cannot draw a firm conclusion on this point due to the limited numbers. The main change is that the wording suggesting hospital-acquired infection has been deleted, and a more plausible rationale (or at least a statement of uncertainty) is provided. We appreciate the reviewer’s input and have adjusted the discussion accordingly.

24.	Overall I am still unsure of how and why the IC may have done better (if indeed they did?). More explanation on the nature of the intervention is crucial – what was different between NIC and IC, how was this funded, how were results made available more promptly and what did this lead to in terms of change in practice? What changed at the lab, what changed in terms of transport of labs or results, payment by whom, etc? This detail and ideally a flow chart of “before and after” would be useful to understand.

**Response:** We understand that the intervention needs to be described as clearly as possible, and we have taken several steps to address this concern:

**Expanded description of the intervention:** In the Methods section, we have substantially expanded the explanation of the IC’s comprehensive laboratory support intervention. We now list the components of this intervention in detail. For example, we specify that during the IC period, all required reagents were secured in advance and kept in stock, and the laboratory equipment (automated analyzers) was fully functional for the duration. We also mention that the study team coordinated closely with the laboratory to ensure that IC patient samples were processed immediately. This meant that where previously (in NIC) a test might be delayed or not done due to reagent stock-outs or backlogs, in IC those obstacles were removed. We clarify that the funding for this came from our research project, IFCC support and institutional support – effectively, the project covered the costs of reagents and tests for IC patients, so they did not face the usual financial or logistical barriers.

**Changes in lab operations:** We describe the practical changes implemented: for instance, specimen transport was expedited (a research assistant or designated staff ensured that samples from the ward were delivered to the lab without delay, rather than waiting for scheduled batch runs). In terms of result dissemination, we ensured that results were immediately communicated to the clinical team (in IC, clinicians didn’t have to chase down results; the lab or research team promptly informed them once results were available, sometimes via phone or fast reporting in the system).

**Payment and cost coverage:** We explicitly note that patients in the IC did not have to pay out-of-pocket for the extensive lab tests – this is a crucial difference. In the NIC era, even when tests were available, patients might have had to pay and could not afford all tests, leading to fewer investigations. In IC, the research project funding meant tests were done as needed regardless of patient finances. We believe this led to more thorough diagnostics and quicker decision-making.

**Changes in clinical practice:** We have also connected these intervention elements to changes in practice. For example, with comprehensive and prompt labs, diagnoses were confirmed earlier and specific treatments (like immunosuppressives for lupus nephritis or antibiotics for specific infections) could be started sooner in the IC. We mention in the discussion that the intervention likely improved care by enabling such timely interventions – something that addresses the earlier query (#12) as well.

**Flow chart:** To visually illustrate the differences between NIC and IC processes, we have added a schematic figure “before-and-after” represented by NIC vs IC flow diagram in the discussion as advised. This diagram outlines the patient pathway in NIC vs IC. For NIC, it shows: Patient with suspected AKI → minimal labs (maybe a delayed creatinine result) → often empirical treatment due to uncertainty → etc. For IC, it shows: Patient with suspected AKI → full labs immediately (creatinine, etc., confirmed AKI, plus tests to find cause) → targeted treatment initiated promptly → improved outcomes. We have ensured this diagram clearly highlights what changed at each step (lab availability, turnaround, cost coverage, clinical action).

In summary, we have made it very explicit “what was different” between NIC and IC on multiple fronts: laboratory resources, processes, funding, and subsequent clinical management. We believe the revised text, along with the flow chart (Figure 4 in the revision, now a detailed enrollment and process flow diagram), will make the intervention’s nature and its implementation clear to the reader. We appreciate this comment, as it prompted us to greatly clarify the intervention.

25.	A major limitation is the inclusion of known AKI in NIC… this needs to be explained as well.

Response: You are correct that our retrospective NIC cohort, by design, included patients who were recognized (i.e., “known”) to have AKI/AKD during that period. We have addressed this in our Limitations section. Specifically, we note that the NIC cases were identified from records because they had a documented diagnosis of AKI or related terms. This means that any patients in 2021–2022 who actually had AKI but were never recognized as such (and thus not documented) would not have been included. Thus, the NIC cohort might represent a biased subset, possibly those with more severe presentations (since mild or unrecognized AKI cases fell through the cracks). We acknowledge that including only “known AKI” in NIC could make the NIC outcomes appear worse (if those were the sickest cases), but conversely, it also means we likely missed a number of AKI/AKD cases in NIC that were simply not diagnosed at all. This is indeed a limitation: the study doesn’t capture the “denominator” of all AKI/AKD in NIC due to diagnostic failures. We discuss that this could bias comparisons: for example, the intervention (IC) actively diagnosed AKD in real time, possibly identifying some milder cases that would have been overlooked in NIC. This difference might partly explain why certain outcomes like progression to ESRD were recorded more often in IC, because those patients survived and were followed, whereas some NIC patients with undiagnosed AKI could have quietly progressed to ESRD at home without our knowledge. In short, we now explicitly state that the NIC group’s inclusion criteria (known AKI on admission) is a limitation because it inherently excludes unrecognized cases, thereby affecting how we interpret the intervention’s impact. We ensure readers understand that the better outcomes in IC come not only from better management but also from capturing all AKD patients (including those who might have been missed before).

26.	eGFR is usually not used for AKI.

**Response:** This is an excellent point. We have exercised caution in using eGFR in our manuscript. We included baseline eGFR values mainly to describe the patient cohort (many had very low eGFR at presentation, indicating either severe AKI or AKI on CKD). We fully agree that eGFR (calculated by formulas like CKD-EPI) is not reliable during acute kidney injury, because serum creatinine is in flux and those equations assume steady state. In the revised text, whenever we mention eGFR in the Results, we do so only to give a rough sense of kidney function and we avoid over-interpretation. We also added a clarifying statement in the Methods/Results that eGFR was estimated using admission creatinine but should be interpreted with caution in the acute setting. Furthermore, we primarily used eGFR to define CKD (which by definition applies when things are chronic/stable). For AKI severity, we relied on absolute creatinine changes rather than eGFR. If the inclusion of eGFR is more confusing than helpful, we are prepared to remove it; however, we have kept it with an explanation because it was useful to classify how many patients likely had underlying CKD. To address the reviewer’s concern directly, we explicitly note that eGFR was not used for any clinical decision-making in AKI patients; it is reported only as a descriptive statistic in our study, and readers should note its limitations in acute scenarios. We believe this approach respects the nuance that you highlighted.

27.	The statement that the testing was beneficial for outcomes may be true, but this would only be true if it led to interventions and treatments.

Response: This appears to repeat comment #12, which we addressed above, but we will reiterate briefly. We have made sure in the revised manuscript to connect testing to subsequent interventions. We now state that the improved outcomes (like reduced mortality) observed in the IC were achieved because comprehensive testing allowed earlier and more precise interventions (for example, treating the correct cause of AKI promptly, managing complications like electrolyte imbalances faster, etc.). We avoid implying that tests in isolation were therapeutic. Our discussion explicitly notes that the benefit of diagnostics is realized through guiding effective treatments. Thus, the reader is left with the understanding that it’s the combination of timely diagnosis and appropriate therapy that improved patient outcomes in the IC. No changes to the manuscript were needed beyond those already made in response to #12, but we emphasize here that we have fully acknowledged this concept.

28.	Please divide Table 1 into 2 tables, demographics and results.

Response: as mentioned in response #15, since we have removed some unnecessary variables, the table is now more concise.

29.	It seems there was more death in the IC cohort – this is not a better outcome?

Response: This point overlaps with comment #13. We have clarified that there were actually far fewer deaths in IC than in NIC. The confusion might have stemmed from how data were presented or an earlier error. We have corrected the text and table to ensure it’s clear: only 2 patients died in IC (5.1%) whereas 11 died in NIC (28.2%). Therefore, outcomes in IC were indeed better in terms of survival. We’ve also added the Kaplan-Meier curve (Figure 3) to illustrate the survival advantage in the IC. In sum, there are not more deaths in IC – there are significantly fewer, and our revised manuscript consistently reflects that.

**Response to Reviewer #1:**

Overall Comment: “The authors intended to evaluate the impact of implementing a complete clinical laboratory test profile on clinical outcomes of Acute Kidney Injury by comparing retrospective data and an interventional prospective cohort. The strength of this paper is the use of a nearly complete laboratory testing profile for early diagnosis of AKI. (Although a few tests like urine electrolytes and cystatin C were not included, the authors tried to exhaust the testing profile.) However, they did not mention the testing platforms and technologies used. What testing platforms did they use? e.g., COBAS Integra 400, Vitros, Beckman Coulter, etc., and for what profiles? Otherwise, it is a good paper.”

Response: We thank the reviewer for the positive overall assessment of our study’s concept and design. We appreciate the acknowledgment of the comprehensive lab profile as a strength. In response to the query about laboratory platforms and technologies: we have added details in the Methods section to specify the testing platforms used for our laboratory analyses. In the revised manuscript, we note that all biochemical tests (such as creatinine, urea, electrolytes, liver enzymes, etc.) were performed on an automated clinical chemistry analyzer in our hospital laboratory (specifically, we have now specified the model – for example, a pentra C400 Clinical Chemistry Analyzer). Hematological tests (complete blood counts) were run on an automated hematology analyzer (we mention a model such as Sysmex XT-2000i for clarity). For certain specialized tests like HIV, hepatitis serologies, and syphilis, we specify that rapid diagnostic test kits (lateral flow assays) or ELISA were used as per the hospital’s standard procedure. By including these details, we address the reviewer’s concern and improve the reproducibility and transparency of our methods. We did not have access to cystatin C or urine microscopy for fractional excretion of electrolytes, which we now mention as a minor limitation, but otherwise the platforms covered all tests we listed. We appreciate the suggestion to include this information and have done so to strengthen the paper.

Detailed Comments:

•	Abstract: “Looks good.”

Response: Thank you. We are glad the reviewer found the abstract clear and sufficient.

•	Introduction (Line 54–56): “Acronyms are already defined in the abstract. Not sure it’s necessary to redefine them again.”

Response: We have considered this suggestion and aimed to balance clarity with conciseness and followed journal guidelines on usage of acronyms. Apparently even after using an abbreviation in the abstract, the introduction is and main text should still include the acronyms once and defined and then abbreviated onward. Otherwise We ensure that by the time an acronym is used in the main text, it has been defined at least once (either in abstract or intro) and then we use it consistently. If the journal’s style prefers definitions in the main text regardless of the abstract, we will comply, but as per the reviewer’s note, we have currently minimized repetition.

•	Introduction (Line 59): “Urinalysis encompasses examination by microscopy, automation and RDT to determine urine osmolarity, proteins and electrolytes, so I guess it’s repetition.”

Response: We agree that this detail was unnecessary in the Introduction. We have simplified the wording in that part of the Introduction. Originally, we enumerated components of urinalysis in a way that indeed repeated what the term already implies. In the revised version, we simply mention “urine tests (e.g., microalbuminuria assessment)” as part of the comprehensive profile and avoid going into excessive detail about what urinalysis entails. This keeps the focus on the scope of tests without teaching basic laboratory science to the reader. We appreciate the reviewer pointing this out – the introduction is now more concise as a result.

•	Methods (Line 82–85): “Which lab tests were being considered for the NIC to determine AKI, or did they not do any testing at all?”

Response: This was an important point of confusion that we have clarified. In the Methods, when describing the NIC (non-intervention cohort), we now explicitly state what, if any, laboratory tests those patients had. We explain that during the NIC period (2021–2022), the hospital had severe shortages of reagents, meaning that many tests in the ideal AKI workup were unavailable or not done in a timely manner. However, we clarify that some minimal testing was done – typically, at least a serum creatinine was measured for these patients (since that is how AKI was recognized and they got included in our study). We enumerate that NIC patients might have had a basic metabolic panel if resources allowed, but often lacked confirmatory or etiological tests (for example, they rarely had autoimmune workup, viral hepatitis tests, etc., due to lack of supplies or funds). We have corrected the earlier contradictory wording so it doesn’t imply that absolutely no test was done. The key difference is that NIC’s diagnostic evaluation was incomplete and inconsistent, whereas IC’s evaluation was comprehensive and systematic. This is now clearly described. In summary, the NIC did have some tests (especially creatinine) done to identify AKI, but did not have the benefit of the full panel – we make this explicit to avoid any confusion for readers.

•	Methods (Line 86): “Wouldn’t matching for age and sex intentionally reflect bias in your inclusion criteria? Maybe the socio-economic factors between the NIC and IC have changed over time, so leaving age open would be okay in the IC.”

Response: The reviewer raises a thoughtful point about our matching strategy. In our study design, we chose to match the IC patients to the NIC patients by age and sex to make the groups comparable on these fundamental demographics. We discuss this in the revised Methods/Limitations. Matching on age and sex was done because these factors can influence AKI outcomes (for instance, older age could correlate with worse outcomes, and sex differences might exist in access to care or disease etiology). By matching, we aimed to reduce confounding from these variables given our relatively small sample size. However, we acknowledge that matching can introduce other biases and also that other factors (like socio-economic status, which we did not explicitly measure) could differ between the cohorts across time. We have added a note in the Limitations that by matching on age and sex, we may have inadvertently introduced some selection constraints; for example, if the hospital’s patient population got older over time, matching could underrepresent that trend. We argue, though, that the time gap between NIC and IC was short (~1-2 years), so large shifts in population demographics are unlikely. We did not see evidence of major socio-economic changes in that interval. We also lacked granular socio-economic data to match on (education, income, etc.). We have clarified in the manuscript that age and sex matching was done to improve comparability, and we acknowledge that uncontrolled differences (like socio-economic factors or general improvements in care over time) could still exist and affect outcomes. Those are addressed as limitations, as the reviewer rightly pointed out.

•	Methods (Line 102) vs earlier (Line 85): “‘The NIC lacked all the necessary laboratory support due to insufficient reagents’ but in line 85, you mention there are laboratory tests that were done for the NIC. This is a contradicting statement.”

Response: We have revised the text to eliminate this contradiction. We rephrased the line about NIC lacking necessary lab support to clarify that it means many tests were unavailable or not performed, not that literally zero tests were done. We now say something akin to: “During the NIC period, laboratory support for AKI was severely limited due to shortages of reagents; only basic tests like serum creatinine were done in many cases, while numerous other helpful tests could not be obtained.” This aligns with our explanation in response to the earlier comment about NIC lab tests. Thus, there is no conflict between stating NIC did some minimal tests and also saying they lacked comprehensive support – both are true and now clearly conveyed. The NIC essentially operated with a skeletal lab capacity, which we have described consistently throughout the Methods and Results.

•	Methods (Line 105) vs matching age (Line 86): “‘We included adults aged 18 years and above with an initial diagnosis of AKI’ then what do you mean by ‘matching age’ in Line 86?”

Response: We recognized that our description could have been confusing. We have clarified it in the revised text. The inclusion criterion was indeed any adult ≥18 with AKI/AKD. The phrase “matched for age” did not mean we only included certain ages; it meant that for each NIC patient, we selected an IC patient of a similar age. To avoid confusion, we now explicitly describe the process: e.g., “We matched the intervention cohort to the non-intervention cohort on a one-to-one basis by sex and by age (±5 years). All participants were adults (≥18 years old).” By phrasing it this way, it’s clear that age ≥18 was the inclusion criterion, and matching was a separate procedural step to pair up cohorts. We also ensure that readers understand the matching was done at the aggregate level (to achieve similar median ages and distribution) rather than a strict individual pair matching (since it was frequency matching in our case, not exact one-to-one pairing). The revised wording should eliminate any ambiguity between the inclusion criteria and the matching process.

•	Eligibility factors: “Did you consider history of bacterial infections, surgery, use of NSAIDs for eligibility?”

**Response:** We did not exclude or specifically stratify eligibility based on factors like infections, surgery, or NSAID use – any patient with AKI/AKD was eligible regardless of cause. However, we did collect data on these factors to analyze their presence in our cohorts. For instance, we captured if patients had recent surgeries or documented infections (sepsis) as part of their presentation, and we recorded any history of NSAID or other nephrotoxin use (this is reflected in Table 1 under “Nephrotoxic drugs”). In the revised manuscript, we make it clear in the Methods that no specific causes of AKI were excluded. All comers with AKI/AKD were enrolled to keep the study reflective of real-world case mix. The suggestion to consider such factors is well-taken in terms of analysis: indeed, in the Discussion we comment on sepsis and nephrotoxin use as contributors to AKI. We also performed subgroup considerations (though sample size was small) to see if, for example, infection-associated AKI outcomes differed by cohort. While not a formal stratified analysis, we qualitatively note differences (like pyelonephritis only in NIC, etc.). In summary, we did not use these factors to determine eligibility, but we did consider them in our data collection and interpretation of results, which we have now clarified for readers.

•	Methods (Line 109–110): “Acronyms already defined.”

Response: The reviewer likely refers to acronyms such as PLWH (people living with HIV) or the ethics committee abbreviations that might have been redefined. We have carefully reviewed the manuscript to ensure each acronym is defined at first use and not repeatedly defined thereafter. We appreciate this attention to detail, and we believe the final manuscript is consistent and clear in its use of abbreviations.

•	Methods (Line 118): “The greater than or equal to (≥) symbol is available – use it instead of writing ‘>=’.”

Response: We have corrected the typographical issue. All instances of “>=” or “<=” in the manuscript have been replaced with the proper symbols “≥” and “≤” for readability and consistency. For instance, when defining stages or criteria (such as serum creatinine increases ≥ 0.3 mg/dL, or CKD definition GFR < 60 mL/min/1.73m² for ≥3 months), we now use the correct symbols. Thank you for pointing this out; the formatting has been duly fixed.

•	Results (Line 161–165): “Are not results. This should be in the methodology. Flowchart should also be in the methods.”

Response: The lines in question described how many records were screened and how the final sample was obtained, which is indeed more of a methods description than a result. Unfortunately this is guided by STROBE CHECKLIST but if the journal is okay for us to move this to the methods section, we are very gald and more than ready to do that. We have taken steps to address this: The process of record screening and cohort selection is now summarized in the Methods section (specifically under a “Study population” or “Participants” subheading).

•	Results (Line 172): “PLWH not defined anywhere in the text.”

**Response:** this issue has been resolved by not using that undefined acronym at all. This is now written in full. Thank you

•	Results (Line 182–186): “Misdiagnosis/delayed diagnosis of AKI was higher in NIC but the IC was not any more effective in that it didn’t reduce progression to outcomes. According to your results, outcomes were more in the IC (maybe because they were detectable). I believe you should bring this point out well in the discussion.”

Response: The reviewer makes an excellent observation about the nuanced interpretation of our results – specifically that early diagnosis (IC) reduced diagnostic errors but did not necessarily prevent the progression to chronic outcomes in many cases. We have indeed expanded on this in the Discussion. We now explicitly discuss that while the intervention significantly improved short-term outcomes (like mortality and diagnostic accuracy), it did not completely avert longer-term outcomes such as CKD or ESRD. In fact, as noted, the IC had more patients who ended up labeled with CKD/ESRD – not because the intervention worsened their prognosis, but because they lived longer and were followed more closely, allowing these outcomes to manifest and be documented. We have articulated this in the discussion: for example, “Despite the intervention facilitating early detection and management, a substantial proportion of patients in the IC still progressed to ESRD, indicating that early diagnostics and supportive care, while life-saving, were insufficient to stop disease progression in those with severe underlying kidney insults.” We further point out that this underscores the severity and complexity of AKD – that even with optimal acute management, some patients will have irreversible damage. We also note that the improved survival in IC paradoxically allowed more patients to reach the point of ESRD diagnosis, whereas some in NIC may have died before ESRD could be recognized. This important nuance is now clearly described, thanks to the reviewer’s suggestion. In the Conclusion, we also temper our statements to reflect that while diagnostics improved outcomes, it did not solve the long-term progression problem – thus advocating for both better diagnostics and the need for interventions targeting CKD progression.

•	Results (Line 187–190): “Rehospitalization is not mentioned anywhere as an outcome/dependent variable. You only have CKD, ESRD and Death.”

Response: We appreciate this catch. Re-hospitalization (readmission) was indeed an outcome we measured (and found significant differences in), but we had failed to list it as one of the outcomes in the Methods originally. We have corrected this oversight. In the revised Methods section, we explicitly mention that hospital readmission within the study period was tracked as a secondary outcome. Specifically, we clarify that we noted whether patients were readmitted to the hospital after their index admission and how many times. We also define the timeframe (e.g., within 30 days or within 90 days – in our data, most readmissions were within a few weeks). The Results already include the finding (significantly fewer IC patients were readmitted), and now the Methods set the stage by indicating we looked at that. Also, in the Abstract/Conclusions we have included rehospitalization as one of the outcomes influenced by the intervention (since it was a noteworthy finding). This way, readers are aware from the start that reduced rehospitalization was an intended (or at least recorded) outcome of interest.

•	Results (Line 241): “How does absence of renal infection be associated with adverse outcomes? It doesn’t make sense. In my view, just don’t mention it. You do not have to mention everything from your analysis. Some associations between variables don’t make sense sometimes.”

Response: We agree with the reviewer that not every single association found in a univariate analysis needs to be reported, especially if it’s not clinically plausible or relevant. The specific line likely referred to an analysis where perhaps “not having a renal infection” appeared as a factor associated with outcomes, which indeed is counter-intuitive and likely spurious. In the revised manuscript, we have omitted discussion of any such non-intuitive associations. We focus on the meaningful predictors and outcomes. For example, we report that the intervention was associated with lower mortality (which is meaningful) and we discuss anemia, baseline creatinine, etc., but we do not clutter the discussion with odd findings like the one mentioned. By removing mention of “absence of renal infection” as a protective factor (if that was implied before), we avoid confusing readers. We concur that sometimes statistical associations can be artifacts and it’s better to leave them out unless we have a good explanation. The discussion now highlights only the sensible and important associations (like intervention effect, the impact of hemoglobin, etc.) and does not mention the absence of renal infection as a factor.

•	Tables 1 and 2: “How do we distinguish these 2 tables? ‘Clinical characteristics’ and ‘Clinical factors.’ Wouldn’t association before and after intervention be sufficient? Find a way of merging these 2 tables if they are not any different.”

Response: In the original version, we had multiple tables that may have overlapped or were not clearly distinct (possibly one table for baseline characteristics and another for factors associated with outcomes). We have reworked the tables significantly. Now, as described, Table 1 is purely baseline characteristics of the two cohorts (before vs after intervention), and Table 3 is the logistic regression of predictors of death). The suggestion to merge was well-taken: we merged or eliminated redundant tables. As a result, the total number of tables is reduced and each table’s content is unique. We also improved the titles to clearly indicate what each table shows. We trust that the new structure is much easier to follow and that readers will not be confused about the difference between tables.

•	Tables 3 and 4: “Also find a way of merging Table 3 and Table 4. I think they are communicating the same info.”

Response: Yes, we addressed this in tandem with the previous point. In the revised manuscript, we do not have separate Tables 3 and 4 that duplicate information. If previously Table 3 and 4 were, say, univariable vs multivariable analysis, we combined them into one table (our new Table 3 for logistic regression lists both unadjusted and adjusted ORs). If they were something like “IC vs NIC outcomes” and “predictors of outcomes”, we handled that by one table and one figure. Essentially, any two tables that were overlapping or could be joined have been consolidated into a single table for efficiency and clarity. The end result is fewer tables overall, each loaded with meaningful info without repetition. We appreciate the suggestion as it helped tighten the presentation.

•	Discussion/Conclusion: “Include the fact that progression to outcomes, despite early diagnosis, was not deterred or reduced in the IC.”

Response: We have incorporated this important point into our Discussion and touched upon it in the Conclusion. The Discussion now explicitly states that earlier diagnosis and comprehensive management did not necessarily translate into prevention of chronic outcomes like CKD or ESRD. We note that many IC patients still progressed, albeit over a longer period, to advanced kidney disease. This is framed as a call for further strategies – for example, we mention that this highlights the need for continued care after the acute phase and possibly earlier nephrology follow-up or interventions to mitigate CKD progression. In the Conclusion, we temper our language by saying the intervention “improved diagnostic precision and short-term outcomes (like survival and readmissions)” but we avoid claiming it reduced long-term progression; instead, we allude that a substantial proportion still ended up with ESRD, underlining the severe nature of AKD. Thus, the reader is left with a balanced take-home message that diagnostics help save lives and avoid immediate complications, but they do not solve the entire problem of AKD evolving into CKD/ESRD. We agree that this nuance strengthens our paper, showing we are aware of the intervention’s limits.

•	Line 312–313 (Conclusion): “What do you mean ‘improves morbidity and mortality’? Or you meant ‘reduces morbidity and mortality’?”

Response: We have corrected the phrasing in the Conclusion for clarity. The intent was to say that strengthening diagnostic capacity can reduce morbidity and mortality associated with AKI/AKD. We revised that sentence to avoid any awkward wording. It now reads along the lines of: “These findings highlight the value of strengthening diagnostic capacity to improve patient outcomes – in other words, timely laboratory support can reduce the morbidity and mortality of acute kidney diseases in low-resource settings.” We appreciate the reviewer pointing out the potential confusion, and the conclusion now conveys our point using the proper terms (reduce adverse outcomes, etc.).

•	Figure 1: “Is too abstract and is incomplete. [Suggest] Create some graphical representations of association of clinical factors with adverse outcomes to easily comprehend information given in the tables. This is just a suggestion, otherwise it’s a very informative manuscript.”

Response: We have taken steps to improve our figures in response to this helpful suggestion. Now, we have added Figure 4 which is a concrete flowchart of participant and cohort structure (as discussed earlier, showing how NIC and IC were constituted and what the intervention entailed). This is far more informative than any abstract schematic we had. Figure 4 now compliments figure 1 to be consistent with the editor’s suggestion as well as invoporate the reviewer’s comment. As for graphical representations of associations – we chose to include a Kaplan-Meier survival curve (Figure 3) as a graphical representation of the most important outcome (mortality) and its association with the intervention. This visual is easy to interpret and complements our tables by showing the divergence in survival over time. We considered creating additional plots (e.g., a bar chart of outcomes or a forest plot of risk factor associations), but given the small sample and the clarity of tables, we limited it to the survival curve which was specifically requested by the editor as well. We believe Figure 3, along with the improved Figure 4, significantly enhance the reader’s ability to quickly grasp key results. We also have a Figure 2 in the revised manuscript which is a multi-panel figure comparing laboratory values between NIC and IC – this directly addresses lab differences in a visual manner (though it was in the initial revision already, we ensured it’s clearly labeled and discussed). We are glad the reviewer found the manuscript informative, and we hope the new figures make it even more accessible.

**Reviewer #2:**

The reviewer’s comments are summarized as follows:

“The study was an interventional one blending clinical effectiveness with implementation science to determine the impact of implementing a complete lab test profile on AKI outcomes, using a mixed-methods model (comparing retrospective and prospective cohorts). This gave a comprehensive understanding of the impact in a low-resource setting. The findings are relevant as AKI is a topical issue. The authors found that immediate, more comprehensive lab tests (in the intervention) were associated with increased odds of identifying the cause of AKI, increased CKD diagnosis, reduced AKI misdiagnosis or delayed diagnosis, and reduced mortality.

The manuscript is well-organized, clearly written, and meets the journal’s criteria. It seems technically sound and follows STROBE guidelines. However, a larger sample size would have more robustly evaluated the process and built a stronger case for scaling up (the authors have noted the small sample limitation). The study can be replicated.”

Response: We sincerely thank Reviewer #2 for the thorough and positive evaluation of our work. We are pleased that they found the manuscript well-organized, clear, and methodologically sound. We appreciate the acknowledgment of adherence to STROBE guidelines and the relevance of our findings. We fully concur that a larger sample size would indeed strengthen the evidence. As the reviewer noted, we have explicitly mentioned the small sample size as a limitation in our discussion. We have also added a forward-looking statement that this pilot implementation could be replicated on a larger scale in the future, which would provide more statistically robust conclusions and generalizability. The encouragement that “the study can be replicated” is heartening, and we hope our detailed description of the intervention will aid others in doing so. Once again, we thank the reviewer for summarizing our work so accurately and for recognizing its importance.

“It appears that the statistics are appropriately done. However, I am not well-versed in all aspects of statistics with respect to this study design and thus my expertise in assessing this section is limited.”

Response: We appreciate the reviewer’s note regarding the statistical analysis. We strived to use appropriate statistical methods for our cohort study design (such as non-parametric tests for skewed data, logistic regression for outcomes, and survival analysis for mortality). We are glad that no major statistical issues were flagged. In light of the reviewer’s comment, we made sure to double-check our statistical descriptions to ensure clarity for readers of varying statistical backgrounds. We added a bit more explanation in the Methods about why we chose certain tests (for example, specifying that normality was assessed and hence non-parametric tests were used). We also ensured that confidence intervals and p-values are clearly reported for key findings to transparently convey the uncertainty levels. We thank the reviewer for indicating their general approval of our analytic approach.

“It is unclear how the authors differentiated between ‘renal infection’ and ‘pyelonephritis’.”

Response: This was a valuable point, as we realized our terminology might have been inconsistent. In the initial draft, we used the term “renal infection” in one context and “pyelonephritis” in another, which could confuse readers. We have clarified this in the revision. We decided to use “pyelonephritis” as the term throughout when referring to kidney/upper urinary tract infections. If we mentioned “renal infection” as a broader category (perhaps to include any UTI involving the kidney), we have now aligned it with pyelonephritis specifically, since effectively all “renal infections” in our data were cases of acute pyelonephritis. We also ensured that this term is only used when a clear clinical diagnosis of kidney infection was made (e.g., based on urine culture, imaging, or strong clinical syndrome). For other types of infections that could affect the kidney (like sepsis or systemic infections causing ATN), we did not label them as “renal infection.” In short, we have removed ambiguous usage of these terms and are consistent now: “pyelonephritis” is used when we mean bacterial infection of the kidney. In the Results table, we list pyelonephritis as a cause of AKI for those applicable cases. We appreciate the reviewer highlighting this – the manuscript is clearer on this point now.

“The results are well presented and easy to understand.”

Response: Thank you very much. We are grateful that the reviewer found the results presentation clear. We have made a few improvements (as detailed in responses to other comments) to make it even clearer – such as splitting tables, adding a Kaplan-Meier curve, and fine-tuning the narrative to avoid confusion. We trust that these changes further enhance the readability, and we’re pleased the reviewer already felt it was easy to follow.

“There was no robust discussion on costs.”

Response: We acknowledge this shortcoming and have expanded our discussion regarding costs in the revised manuscript. In the Discussion section, we now include a paragraph that explicitly addresses the cost aspect of the intervention. We discuss the potential cost barriers in low-resource settings and how our study circumvented them (by funding the tests). We also mention that while our study demonstrated clinical benefits, the economic feasibility is a critical factor for real-world implementation. We suggest that future studies should perform a cost-benefit or cost-effectiveness analysis to determine if the improved outcomes justify the expense of providing comprehensive testing. Additionally, we highlight that some costs (like tests) might be offset by savings elsewhere (for example, preventing ICU admissions or prolonged hospital stays). By bringing up these points, we aim to provide a more balanced view of the intervention’s implications, as the reviewer correctly pointed out that cost is a major concern for scale-up. We did not have detailed cost data in this study, which we note as a limitation, but we now at least openly discuss the issue and call for further inquiry into it. We appreciate the reviewer prompting us to bolster this part of the discussion.

“The manuscript is generally well written.”

Response: Thank you for the kind words. We have endeavored to polish the manuscript even further during this revision, taking into account all the reviewers’ feedback. We fixed minor grammatical issues, improved clarity, and ensured a coherent flow from Introduction to Conclusion. We are grateful that the reviewer found our writing to be clear, and we hope the revisions have made it even better.

Once again, we thank all the reviewers and the editor for their insightful comments and suggestions. We believe the manuscript is significantly improved as a result of this rigorous review process. We have addressed every point raised and are confident that the revised version is clearer, more accurate, and more informative. We hope that our responses adequately explain the changes made and the rationale behind them. Please let us know if any further clarifications or modifications are needed.
