# Supplementary material for: Reducing mortality in acute kidney disease through comprehensive laboratory support: Findings from the ICLATA hybrid implementation study in Zambia
Source: PLOS Glob Public Health. 2025 Oct 14;5(10):e0005342. doi: 10.1371/journal.pgph.0005342 (PMC12520337; doi:10.1371/journal.pgph.0005342)
Supplement: Attachment — Submitted filename: RESPONSE TO Reviewer 2.docx [file pgph.0005342.s005.docx]

Reviewer #2: This study addresses an important problem in low-resource settings: diagnostic capacity as it concerns acute kidney disease. The manuscript is clearly written and fairly easy to understand. It represents a clear improvement over the previous submission.
The methodology section is well described, more scientifically-sounding and the flow is much improved. The statistical methods used are appropriate.
The representation of the results is much improved and clearer to the reader.

**RESPONSE: We sincerely thank Reviewer #2 for their thoughtful and constructive feedback, which has significantly improved the clarity and precision of our manuscript. We have carefully addressed each point raised, as detailed below.**

**Reviewer Comment 1:** Table 1 should be retitled appropriately. The way it is written currently sounds as if it is the same set of study participants who were tested before an intervention, and again after the intervention. This is not the case.

**RESPONSE: We agree with the reviewer that the original title was ambiguous and could be misinterpreted. We have revised the title of Table 1 to clearly indicate that it compares two distinct, matched cohorts from different time periods. New title reads “Table 1. Baseline sociodemographic and clinical characteristics of the non-intervention and intervention cohorts.”**

Reviewer Comment 2: In the row referring to suspected underlying cause of AKD, all the identifiable causes should be listed first. The ‘Other’ should be placed in the last row, below the others.

**RESPONSE:** **This is an excellent point for improving the logical flow of the table. We have reordered the rows in the "Suspected Underlying cause of AKD" section to list all specific causes first, with the "Other" category moved to the final row.**

Reviewer Comment 3: Table 2 is wrongly titled. These are laboratory parameters, and not sociodemographic or clinical factors.

**RESPONSE: The reviewer is correct. We apologize for this error. The title has been changed to accurately reflect the content of the table, which presents factors associated with mortality from a logistic regression analysis that included laboratory parameters. The new title reads “Table 2. Factors associated with mortality in univariable and multivariable logistic regression analyses.**

Reviewer Comment 4: Figure 4 can be better titled. It appears to be a comparison and the figure title should reflect that.

**RESPONSE:** **We thank the reviewer for this suggestion. We have revised the title of Figure 4 to more explicitly state that it is a summary comparison of key outcomes between the two cohorts.**

**New title “Figure 4. Comparison of key clinical outcomes between the non-intervention (NIC) and intervention (IC) cohorts.**

Reviewer Comment 5: The discussion and conclusion sections are well balanced and adequately supported by the data. It was stated (page 17, line 464) that many of the IC patients had underlying CKD. The determination of the ‘underlying CKD’ is unclear.

RESPONSE: We appreciate the reviewer's positive feedback on the discussion and conclusion. We also thank them for pointing out the need for clarity on how underlying CKD was determined. This was based on a combination of clinical history, previous laboratory records (if available), and the diagnostic workup performed during the study. We have added a clarifying sentence to the Methods section to explicitly state this. Furthermore, we have slightly refined the sentence in the Discussion to improve clarity.

There are quite a number of limitations. However, they are clearly stated. The abstract accurately conveys what has been found.

**RESPONSE: We thank the reviewer for acknowledging the clarity of our limitations section and the accuracy of the abstract.**
